# PERPENDICULAR DOUBLE-PLATE FIXATION WITH LOCKING SYSTEM FOR ACROMION PEDICLE FRACTURE

**DOI:** 10.1590/1413-785220162402141691

**Published:** 2016

**Authors:** Junkun Zhu, Zhijun Pan, Rongzong Zheng, Shuhua Lan

**Affiliations:** 1. Zhejiang University, the Second Affiliated Hospital, Orthopedics Department, Hangzhou, Zhejiang Province, China; 2. Lishui Central Hospital, Orthopedics Department, Lishui, Zhejiang Province, China

**Keywords:** Fractures, bone, Acromion, Fracture Fixation, Internal

## Abstract

**Objective:**

: To describe the surgical technique and preliminary clinical outcomes in a series of open reduction internal fixation of basal acromion process fractures applying a double-plating technique.

**Methods:**

: Nine consecutive patients, mean age 33.4 years old (range, 23-61 years old) with unilateral acromion fracture (Type 3 AO/OTA) with more than 1cm displacement who underwent fixation utilizing a locked double-plating technique, were evaluated on average at 7.8 months (range, 3-15 months) for outcomes related to pain, shoulder function, and surgical complications.

**Results:**

: Eight patients recovered with complete radiographic union and favorable shoulder function. One case failed to be fully evaluated for more than 3 months follow-up. The overall scores of Constant, Shoulder Pain and Disability Index (SPADI) and DASH for the eight patients reviewed were 91.9± 6.31, 3.11± 3.79 and 5.2± 6.35, respectively. No post-operative infection or surgical hardware irritation was identified at final follow-up of these eight patients.

**Conclusion:**

: While more evidence is needed to justify its advantages over traditional implants, perpendicular double-plate with a locking system may be indicated for acromion pedicle fracture treatment, since it performed well for fracture healing and joint function rehabilitation. Level of Evidence IV, Therapeutic Study.

## INTRODUCTION

Fractures of the acromion and scapular spine have recently raised great interest among joint surgeons, for the significant morbidity relative to reverse shoulder arthroplasty. However, the state of the art about acromion fracture, as a result of primary injury events, drew little attention in academia. Kim et al.^1^ argued that early surgical procedure should be considered even in cases of OTA type IC acromion fractures for active young patients. As part of the superior shoulder suspensory complex, the acromion bears continuous axial bending stress, contributing to displacement of even initially non- or minimally-displaced fractures.^2^ Given that the major stress loading on the acromion comprises anterior-inferior, posterior-inferior and lateral vector components, we hypothesized that a three dimensional (3D) fixation technique featuring perpendicular double-plate with a locking system would provide multiple directional stability. Generally, tension band combining K wire has been proved fairly stable; nevertheless, the associated hardware irritation drastically impairs functional movement and quality of life.^3^ Hence, contemporary surgeons prefer reconstruction plate and lag screw to address this issue.^4^ The uniqueness anatomy of acromion pedicle structure accounts for more attempt at cost effective surgical resolution on facilitating joint function, as well as bone union. This study yields relevant insight to the 3D stable fixation strategy and associate rehabilitation tactics regarding pedicle acromion fracture.

## METHODS

Nine consecutive patients (treated in the period 2003-2011) undergoing open reduction and internal fixation (ORIF) surgery for acromion pedicle fracture utilizing perpendicular double-plate were observed in this study. The procedures followed in this study were closely in accordance with the Helsinki Declaration of 1975, as revised in 2000. We obtained the approval of the Institutional Ethics Committee and nine cases of the approval protocol with signed Informed Consent forms. Routine x-ray, intra-operative fluoroscope, 2D-CT and 3D-CT reconstruction images were documented for surgical assessment. The inclusion criterion was significantly displaced (≥ 1 cm) acromion fractures (Type3 AO/OTA). Basically, subjects with surgery contradiction for systematic abnormality are unsuitable to be enrolled. Moreover, patients unable to abide supervised post-operative rehabilitation exercise program following surgical protocol, those with severe cerebrospinal trauma, who are supposed to hardly come around immediately after operation, and those unwilling to comply with more than 12 weeks follow-up surveillance were excluded. As a result, 2 of 11 patients who accepted the relevant surgery failed to participate in the series on account of the standard for exclusion. The nature and purpose of the operation, in line with their risks and benefits, as well as the possibilities of complications and the alternatives to this operation, had been fully explained to all the subjects included before they signed the Consent forms. All of the operations were performed by the same surgeon (SHL) in our level-1 trauma center. 

**Figure 1.**
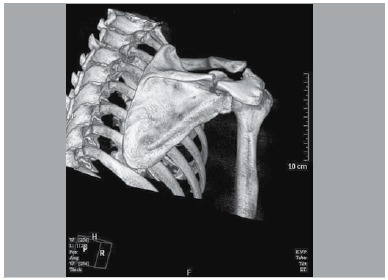
The acromion pedicle fracture illustrated by 3D CT reconstruction.

The demographics of our patients are summarized in Table 1. The routine AP x-ray, intra-operative fluoroscopy, 2D-CT and 3D-CT reconstruction images were documented for surgical assessment. The mechanism of injury included motor vehicle collision (MVC), bicycle accident and blunt trauma. (Table 1) Of the nine patients, six cases underwent ORIF within 3 days of injury, and the remaining cases were treated operatively between day 3-10. As Figure 1 shows, the diagnosis of acromion pedicle fracture was established via pre-operative 3D CT scan which was applied for all candidate participants. The members of another team were responsible for associated assessment, based on questionnaire and physical examination every two weeks until three months follow-up. After that, the patients were required, according to the protocol, to return monthly for evaluation. We also prescribed monthly AP and oblique x-rays to each subject postoperatively. If the x-ray image indicated union signs, we recommend a CT scan for further confirmation. Post-operative function outcomes were evaluated in terms of Constant, DASH (Disabilities of the Arm, Shoulder and Hand) and the Shoulder Pain Disability Index (SPADI). No postoperative infection or implant failure were noticed in our patient group. One patient was additionally diagnosed with a rotator cuff tear and underwent arthroscopic repair two weeks after the fracture surgery. He was not allowed to commence active shoulder movement until three months afterwards, starting from rotator cuff reconstruction operation and he achieved good function, eventually.

**Table 1.** Demographic data of acromion pedicle fracture patients included in the study.

**PatientNº**	**Gender**	**Age(years old)**	**Injury category**	**Concomitant shoulder lesion**	**Healing time (months)**	**Follow-up duration (months)**
1	F	29	Fall	No	4	4
2	M	58	MVC	Glenoid fracture	5	8
3	M	35	MVC	Clavicle fracture	6	9
4	M	23	Blunt strike	No	4	15
5	F	61	Fall	No	3	5
6	M	27	MVC	Rotator cuff tear	4	12
7	M	42	MVC	No	3	5
8	M	26	Bicycle slip	No	4	9
9	F	33	MVC	No	-	3

## SURGICAL TECHNIQUE

**Figure 2.**
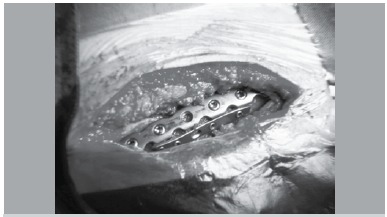
Double-plate perpendicular fixation intra-operative photo.

A posterior-lateral oblique approach was exploited to reduce all the included acromion pedicle fractures. In one case, a concomitant glenoid fracture was identified and treated with a minimally-invasive inverted-L approach, as described by Noort et al.^5^ For the remaining cases, the oblique approach to the acromion was made centering over the fracture along the posterior scapular spine toward the distal part of acromion appropriately. (Figure 2) We utilized the trapezius supraspinatus interval to access deep tissue. The trapezius was dissected off the acromion with supraspinatus retracted cephalad to expose the fragments.^6^ The fracture was reduced with a pointed clamp under direct visualization. The supra-scapular nerve was identified clearly to make sure that it was not pinched between fragments during the fixation process. If the nerve had been entrapped previously, associate exploration procedure would be necessary. It was essential to retract supra-scapular nerve with rubber string to scrutinize when the fixation had been completed. The anterior-superior acromial cortex was fixed with a 2.7mm locking plate system combining 3-4 screws (Synthes) then followed by the utilization of 3.5 mm locking plate system with 4-5 screws (Synthes) on the posterior superior acromion. Thus, perpendicular internal fixation was created to provide augmented resistance against the multiple directional strains that tear the fragments apart. (Figure 2) It is recommended to keep the locking screws engaging but not penetrating the contralateral cortex to minimize the hazard of sub-acromion impingement and distal supra-scapular nerve compromise.

## REHABILITATION PROTOCOL

Phase I (0 - 3 weeks) Post operation acute course

Begin elbow, wrist and hand active range of motion (ROM) followed by shoulder CPM for 30-60° abduction on Day 1;

Sling application when not doing physical therapy with vigorous pain control medication and electrotherapy;

Start moderate pendulum exercises on Day 3;

Start active abduction (15-20°) without any painful movement on Day 7 increasing 10° per day if there was no pain complain;

Gradually increase passive abduction ROM up to 180° over time;

Ensure the active ROM did not exceed 90° during Phase I;

Prescribe a home exercise program combining shoulder blade squeezing and wall crawling 3-5 times per day for 30 min after hospital discharge.

Phase II (3 - 6 weeks) Post operation sub-acute course

Perform assisted forward elevation (FE) over head; 

Perform pulley exercises when tolerated;

Perform isometric exercises for IR, ER, extension, and abduction.

Phase III (7 weeks - 3 months) Returning to normal activity course

Start active shoulder abduction to full ROM in supine position;

Progressively transit from supine to erect position while performing full active supine ROM;

Start light weight bearing (< 2 lb) ROM in all directions when x-ray show healing signs.

## RESULTS

**Figure 3.**
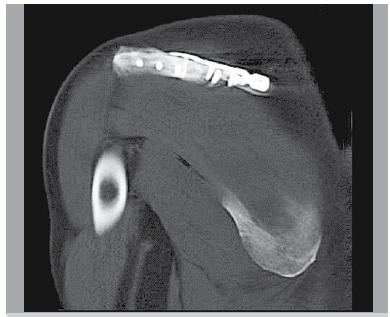
The 2D CT image of radiographic union at three months follow-up.

**Figure 4.**
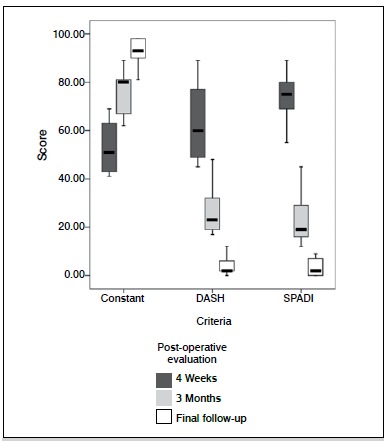
Post-operative clinic data summarized in box-plot diagram processed by SPSS version 15.0.

Eight patients were followed to union, while one patient was lost to further follow-up after three months. In the eight patients evaluated, fracture union was achieved based on radiographs and physical exam. As shown in Figure 3, a patient showed healing through 2D CT image at three months follow-up. The scores of Constant, Shoulder Pain and Disability Index (SPADI) and DASH were 91.9 ± 6.31, 3.11 ± 3.79 and 5.2 ± 6.35, respectively, based on final follow-up findings. Associated clinic outcome in terms of multiple criteria is illustrated in Figure 4. 

We found one case of intra-operative supra-scapular nerve entrapment in the fracture line before prompt neurolysis procedure and the concomitant suprascapular nerve palsy, which resolved within three months. All but one case with comminuted glenoid fracture recovered to favorable shoulder range of movement with 143 ± 15°abduction and 137 ± 19° forward elevation. The patient developed moderate shoulder stiffness with no more than 90° abduction and forward elevation at eight months follow-up. Five cases presented pulmonary contusion and another two cases mild traumatic brain injury besides acromion fracture. Little correlation between the mentioned visceral comorbidities and the fracture prognosis was detected, in accordance with our observation. 

## DISCUSSION

A multitude of researches focused on correlation between categories and prognosis regarding scapular fracture,^7^ however pretty few documents have elaborated on innovations of fixation arrangement about acromion fracture, since the associate establishment of AO/OTA classification. Owing to suspensory function defect, the subsidence of distal acromion fragment definitely undermines shoulder movement. Hardly considered identical to the scapular spine, the acromion pedicle base gets less buttress from scapula body. At the time of injury, excessive impact conducts along acromion long axis to the level of pedicle, where stress accumulates abruptly, therefore, the area is susceptible to fracture, comparatively. In a sense, the junction of scapular spine and acromion can be regarded as posterior scapular "surgical neck". The anatomical property and biomechanical circumstance of the structure demands more insights on the ORIF strategy. 

In the aspect of mechanical configuration, perpendicular planar locking fixation sets a stage for resistance against deforming stress toward variable directions. Even so, the resulting stress shielding is believed to undermine critical healing mechanism.^8^ Additionally, fracture end on the tensile side would be put at risk of non-union, due to strain rate problem. Likewise, the disturbed blood supply seems troublesome for soft tissue detachment. Despite all that, so far the rationale of locking plate has been mostly concluded from experiments with long bones. The results demonstrated in Figure 3 may shed a new light on this issue. Regarding the particular anatomy structure, the double-locking plate outfit warrants sufficient stability, as well as it avoids excessively rigid or flexible implant construction. The newly introduced technique of far cortex locking (FCL) screw is developed from the fact that locking the cortex contralateral to the plate provides more suitable biomechanics for fracture healing, according to preliminary animal trials.^9^ It may partially help expound the context that perpendicular plates inherently provide similar far cortex stability, due to its geometric characteristics. Moreover, intact posterior-inferior periosteum retains adequate vascularization as a healing requisite that is able to quell concerns about atrophic non-union. Though it needs further ergonomics probes on the double-plate performance, this technique is worth bidding for large scale clinic verification. 

Some studies suggested that double-cortex engagement for locking screw puts up considerable superiority over single cortex.^10^ On the other hand, the more aggressive screw choice leads to higher intra-operative risk. According to the surgeons, it appears somewhat challenging to keep the screw tip precisely engaged to the contra-lateral cortex. Consequently, if an operation team supervisor intended to avert spike associated troubles, the mechanical property of double-plate locking system would provide an extra advantage, because it allows for relatively conservative screw insertion, without compromising fixation reliability. 

Last but not least, the technique renders an improved level of efficiency for rehabilitation exercises. An ideal ORIF treatment should not be expected to merely guarantee "healed" bone, but also effectively improve recovery of the patient's daily activities. Most of included patients had good compliance during the rehabilitation courses, after hospitalization. Owing to restore 3D dynamic stability, physical therapists are able to adopt a more active protocol, outweighing the concern about hardware failure. Our team came up with a bolder plan covering prompt movement and accelerated transition of passive-active modalities. The renovated sessions have been preliminarily validated for effectiveness and safety by the series of observation. 

The main drawback of our study is the absence of comparison with a similar group of patients treated with traditional single plate fixation. Due to the sporadic cases of acromion pedicle fracture, we were incapable of conducting a respectable cohort comparison trial. Actually, so far there has been no published RCT clinic article regarding acromion pedicle fracture in major academic literature databases. Our intention was to bring forward a feasible option to the acromion pedicle fracture surgery, as well as to describe a relevant fixation technique. Moreover, our report contributes for building clinic evidence to update post-operative rehabilitation scheme in acromion pedicle fracture cases. The encouraging results of our study are supposed to reinforce physical therapy staff to play a more active role regarding efforts to promote postoperative recovery of the patients.
